# Fractional exhaled nitric oxide and blood eosinophils in relation to lung function, allergy and nasal polyps in asthma

**DOI:** 10.1016/j.jacig.2026.100739

**Published:** 2026-06-01

**Authors:** Reshed Abohalaka, Lauri Lehtimäki, Selin Ercan, Daniil Lisik, Saliha Selin Özuygur Ermis, Pinja Ilmarinen, Teet Pullerits, Helena Backman, Madeleine Rådinger, Bright I. Nwaru, Hannu Kankaanranta

**Affiliations:** a Krefting Research Centre, Department of Internal Medicine and Clinical Nutrition, Institute of Medicine, Sahlgrenska Academy, University of Gothenburg, Gothenburg, Sweden; b Allergy Centre, Tampere University Hospital, Tampere, Finland; c Faculty of Medicine and Health Technology, Tampere University, Tampere, Finland; d Department of Public Health and Clinical Medicine, Umeå University, Umeå, Sweden; e Department of Respiratory Medicine, Seinäjoki Central Hospital, Seinäjoki, Finland

**Keywords:** Blood eosinophil count, fractional exhaled nitric oxide, Feno, asthma, allergic sensitization, allergy, lung function

## Abstract

**Background:**

Type 2 inflammation plays a central role in asthma, but blood eosinophil count (BEC) and fractional exhaled nitric oxide (Feno) levels may reflect different type 2 pathways. Their associations with different asthma characteristics in unselected populations remain unclear.

**Objectives:**

To examine how BEC and Feno, independently and synergistically, are related to lung function, allergy, and nasal polyps in a population-based asthma cohort.

**Methods:**

Data were analyzed from 1567 participants with physician-diagnosed asthma in the West Sweden Asthma Study. Lung function, bronchodilator responsiveness, and presence of allergy and nasal polyps were evaluated across categories of BEC and Feno labeled as low, medium, or high using various cutoffs. Associations were assessed using linear and logistic regression models in participants who were inhaled corticosteroid (ICS)-naive and ICS-treated separately.

**Results:**

Of participants, 49% (n = 767) were on ICS treatment at the time of examination. Among those who were ICS-treated, BEC, independently of Feno, was associated with reduced FEV_1_ and forced vital capacity percentages, higher nasal polyps and clinical allergy, and together with Feno contributed to bronchodilator responsiveness. Interaction effects between Feno and BEC were observed primarily for FEV_1_ and forced vital capacity percentages, at high range of cutoff values. In contrast, among participants who were ICS-naive, neither Feno nor BEC showed independent associations with outcomes; instead, their synergistic effect was associated with lower FEV_1_ and forced vital capacity percentages and a higher prevalence of clinical allergy, with consistent effects across a range of cutoff values.

**Conclusion:**

BEC and Feno reflect distinct yet complementary aspects of type 2 inflammation in asthma. These findings support the joint use of BEC and Feno for obstructive airway diseases phenotyping.

Type 2 (T2) inflammation is the main inflammatory mechanism in asthma, found in about 50%-70% of individuals with asthma.^1^ Active T2 inflammation can lead to repeated exacerbations in moderate-to-severe asthma,^2^ increased asthma morbidity,^3^ increased use of oral corticosteroids,^4^ and permanent lung function decline ^5^ characterized by prebronchodilator FEV_1_ decline.

Blood eosinophil count (BEC) is an important indicator of T2 inflammation. It is associated with the degree of IL5 activity both systemically and in the airways. Anti-IL5 treatments greatly lower BEC.^1^ Although BEC correlates well with serum IL5, it does not correlate well with measures of local airway inflammation, such as sputum cytokines (IL4, IL13, IL33, and TSLP) in those with severe asthma using inhaled corticosteroids (ICS).^6^ On the other hand, fractional exhaled nitric oxide (Feno) is driven by IL13 and reflects IL13 activity in the airways.^7^ Therefore, Feno is significantly reduced with anti-IL13 and anti-IL4R agonist treatments, but less so with anti-IL5 therapy.^8^^,^^9^ In contrast to BEC, Feno correlates with many inflammatory components of the airway T2 immune response in sputum in those with severe asthma using high-dose ICS.^6^

Both BEC and Feno play important roles in the assessment of asthma, including definition of its phenotypes, severity, and evaluation of treatment choices.^10^ Although the Global INitiative for Asthma recommends using BEC and Feno to identify patients with asthma and T2 inflammation,^10^^,^^11^ recent research proposes that BEC and Feno reflect different aspects of T2 inflammation in patients with severe asthma. Feno reflects airway inflammation and the biological signals driving eosinophils into the lungs. Meanwhile, BEC reflects the overall reservoir of eosinophils and circulating IL5 in the body.^12^ This theory means that while BEC and Feno are each strong predictors of asthma events and exacerbations,^13^ they may have synergistic roles in predicting exacerbations in patients who have asthma and are on ICS therapy.^6^^,^^12^^,^^14^ However, it is still unclear how BEC and Feno, independently or synergistically, relate to lung function and allergy in ICS-naive and ICS-treated general asthma populations. Therefore, we aimed to assess how BEC and Feno are related to lung function, allergic sensitization, and nasal polyps in ICS-naive and ICS-treated population-based asthma samples.

## Methods

Full description of the methods can be found in this article’s Online Repository (available at www.jaci-global.org).

### Study area and population

**Fig 1:**
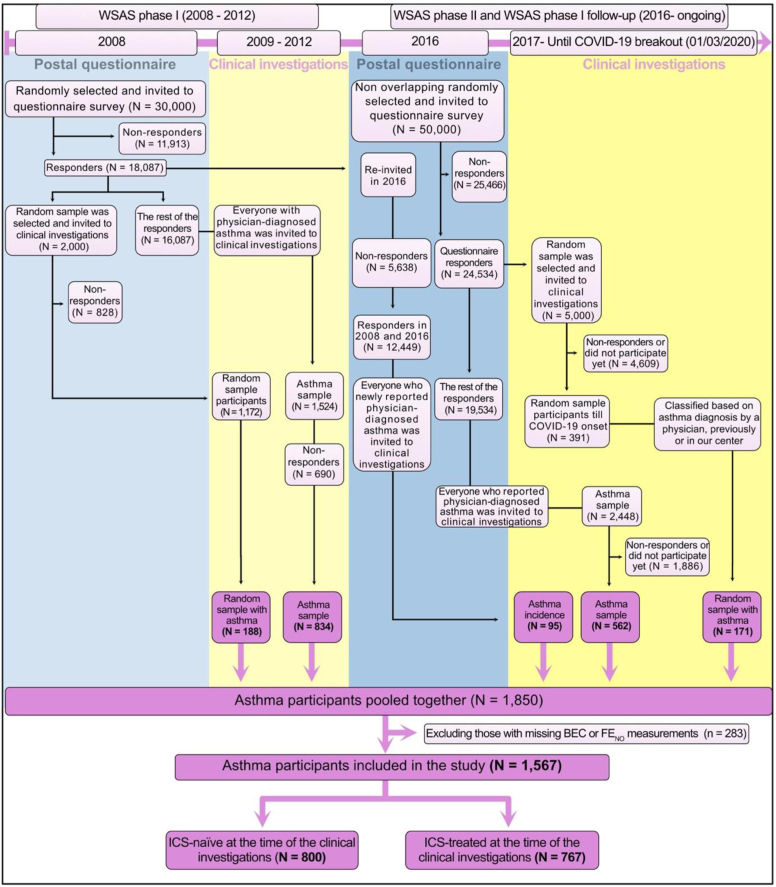
Flowchart of WSAS recruitment and selection of subsample for the present study (participants with physician-diagnosed asthma and clinical examination data, including BEC and Feno measurements, examined between January 1, 2009 and March 1, 2020). *COVID*-*19*, coronavirus disease 2019.

The WSAS (West Sweden Asthma Study), a large population-based cohort study including participants with and without asthma, has been described in detail previously.^15^, ^16^, ^17^, ^18^, ^19^ From the total cohort, a total of 1567 individuals with physician-diagnosed asthma and complete T2 biomarker data were recruited between 2009 and 2020. Among these, 767 (49%) were ICS-treated at the time of recruitment. ICS use was defined as an affirmative response to the question: “Do you currently use, or have you used in the past 12 months, ICS treatment, either alone or in combination?” (Fig 1).

### Clinical examinations

The examinations included, but were not limited to, blood cell quantification, skin prick testing (SPT), specific IgE (sIgE) level assessment, spirometry, and measurement of height and weight.

### Assessment of BEC

BECs were determined as previously described^11^ using standard procedures at Sahlgrenska University Hospital (Gothenburg, Sweden) with ADVIA 2120i Hematology System (Siemens Healthineers, Erlangen, Germany), and are reported as the number of cells per microliter.

### Measurement of Feno

Feno was assessed using an electrochemical device (NiOX VERO, Aerocrine, Morrisville, NC), as previously described,^20^ adhering to the latest American Thoracic Society/European Respiratory Society guidelines.^21^^,^^22^

### Measurement of spirometry

Spirometry tests were done using the MasterScope spirometer (Jaeger, Höchberg, Germany) following American Thoracic Society/European Respiratory Society Taskforce guidelines.^23^^,^^24^ Reference values from the Global Lung Initiative were used to calculate the FEV_1_ as a percentage of predicted normal (FEV_1_%) and forced vital capacity (FVC) as a percentage of predicted normal (FVC%) (25).^25^

### Assessment of allergic sensitization and clinical allergy

Allergic sensitization was assessed through the determination of sIgE levels and/or SPTs. Quantification of IgE levels was executed using the ImmunoCAP system (Phadia AB, Uppsala, Sweden). The SPTs comprised a standard panel of 11 aeroallergens (ALK-Abelló, Hørsholm, Denmark). Clinical allergy was defined by the presence of allergic sensitization (positive SPT or sIgE to any allergen), and self-reported allergic symptoms attributable to the same allergen family.

### Statistical analyses

In our cohort characteristic analyses, comparison of means was conducted using Student *t* test after checking for normality, and comparison of proportions were conducted using chi square test.

### Regression analysis

#### Exposure variables

Participants were categorized based on Feno into 3 groups (≤20, 20-50, and >50 parts per billion [ppb]) and on BEC into 3 groups (≤150, 150-300, and >300 cells/μL) labeled as low, medium, and high. Both biomarkers were entered into the models as categorical factors. An interaction term (Feno × BEC) was included to evaluate joint associations.

#### Outcome variables

Outcomes were analyzed as either continuous or binary variables. Continuous outcomes included lung function parameters (FEV_1_%, FVC%, and FEV_1_/FVC ratio). Binary outcomes included having nasal polyps, clinical allergy, or positivity to bronchodilator responsiveness test defined as ΔFEV_1_ ≥ 200 mL and reversibility ≥12% from baseline FEV_1_.

For continuous outcomes, linear regression models were fitted with Feno and BEC categories and their interaction as independent variables. All models were adjusted for age, sex, body mass index (BMI), and smoking. Adjusted subgroup means and 95% CIs for each Feno-BEC combination were estimated using marginal means derived from the fitted models, with covariates held at their mean values.

For binary outcomes, logistic regression models with the same exposure structure and covariate adjustments were used. Adjusted predicted probabilities were obtained for each Feno-BEC combination, and odd ratios (ORs) with 95% CIs were calculated using the Feno ≤20 ppb and BEC ≤150 cell/μL group as the reference. Regression *P* values were extracted for the main effects of Feno and BEC as well as for their interaction. Statistical significance was defined as *P* < .05 for main effects and interaction terms.

#### Visualization

Results were visualized using heatmaps. For continuous outcomes, each tile represents the crude mean or adjusted mean and 95% CI for a given Feno -BEC subgroup. For binary outcomes, tiles display proportions or adjusted ORs with corresponding 95% CIs. Color gradients were scaled to reflect the clinical direction of effect, with *green* indicating more clinically favorable outcomes and *red* indicating less clinically favorable outcomes.

#### Sensitivity analyses for Feno and BEC cutoffs

To assess the robustness of our findings against the choice of biomarker cutoffs, we performed a systematic grid-based sensitivity analysis. Instead of relying solely on the conventional cutoffs for Feno and BEC, we generated a range of alternative thresholds: Feno thresholds were varied from 15 to 55 ppb in increments of 5 ppb. BEC thresholds were varied from 100 to 350 cells/μL in increments of 10 cells/μL. For each cutoff combination, Feno and BEC were recategorized into low, intermediate, and high groups, with the high category defined as values exceeding twice the selected cutoff. Linear and logistic regression models adjusted for age, sex, BMI, and smoking exposure were refitted for the continuous and categorical outcomes at each cutoff combination, respectively. Regression coefficients, ORs, and *P* values for Feno, BEC, and their interaction were extracted and summarized across the cutoff grid to evaluate the stability of main and interaction effects. All statistical analyses were done separately on participants who were ICS-naive and those who were ICS-treated and executed using R software (version 4.5.0; R Foundation, Vienna, Austria).

## Results

### Characteristics of WSAS participants based on BEC and Feno cutoffs

Participants who were ICS-treated by the time of examination (n = 767, 49%) were older; mostly female; had lower lung function; and had higher BMI, reversibility, and smoking history than the participants who were ICS-naive (n = 800, 51%) (see Table E1 in this article’s Online Repository at www.jaci-global.org). The combinations of sets of cutoffs (20 and 50 ppb for Feno, 150 and 300 cells/μL for BEC, respectively) significantly separated participants by sex, smoking history, pre- and postbronchodilator FEV_1_/FVC, bronchodilator response (%), positive bronchodilator responsiveness test, clinical allergy, and ICS treatment (Tables I and II). However, the combination of those with Feno > 50 ppb and BEC ≤ 150 cells/μL was not common among participants (n = 17) (Table II).

**Table I:** Participants characteristics, lung function, and allergy stratified by cutoffs of Feno (20-50 ppb) and BEC (150-300 cells/μL) (N = 1,567)

Feno (ppb)	≤20		*P* value	>20		*P* value	≤50		*P* value	>50		*P* value
BEC (cell/μL)	≤150	>150		≤150	>150		≤300	>300		≤300	>300	
Demographics												
No. participants	417 (26.6)	445 (24.4)		184 (11.7)	521 (33.3)		1214 (77.5)	189 (12.1)		93 (5.9)	71 (4.5)	
Age (y)	47 ± 15.5	48.9 ± 15.5	.069	50.9 ± 15.6	51.4 ± 15.5	.706	49.1 ± 15.6	49.7 ± 15.9	.644	50.4 ± 15.5	53.3 ± 14.9	.233
Sex (female)	122 (29.3)	160 (36)	.043	98 (53.3)	249 (47.8)	.234	459 (37.8)	83 (43.9)	.128	51 (54.8)	36 (50.7)	.713
BMI (kg/m^2^)	26.5 ± 5.1	27.4 ± 5.4	.009	26.4 ± 4	26.7 ± 4.4	.445	26.8 ± 4.9	27.1 ± 5	.536	26.6 ± 3.8	25.8 ± 4.3	.246
Never smokers	217 (52)	197 (44.3)	.022	107 (58.2)	288 (55.3)	.556	610 (50.2)	101 (53.4)	.473	56 (60.2)	42 (59.2)	.999
Current smokers	51 (12.2)	70 (15.7)	.168	9 (4.9)	27 (5.2)	.999	121 (10)	27 (14.3)	.095	6 (6.5)	3 (4.2)	.733
Pack-year	14.8 ± 15.9	14.4 ± 13.6	.785	14.5 ± 13.9	12.8 ± 14.1	.383	14.3 ± 14.2	15.4 ± 17.6	.595	8.9 ± 8.5	11.5 ± 13.8	.401
Lung function												
Prebronchodilator FEV_1_%	94.0 ± 17.2	89.9 ± 17.4	<.001	92.2 ± 17.6	89.9 ± 18.2	.135	91.7 ± 17.5	89.8 ± 18.1	.163	89.6 ± 17.3	89.2 ± 20.5	.883
Prebronchodilator FVC%	99.9 ± 14.5	97.1 ± 14.9	.006	99.2 ± 13.9	98.5 ± 15.9	.569	98.6 ± 14.7	97.2 ± 16.6	.259	99.6 ± 14.1	98.7 ± 17.3	.699
Prebronchodilator FEV_1_/FVC	0.76 ± 0.09	0.74 ± 0.09	.007	0.74 ± 0.09	0.72 ± 0.09	.140	0.74 ± 0.09	0.74 ± 0.08	.427	0.71 ± 0.09	0.71 ± 0.09	.891
Postbronchodilator FEV_1_%	98.7 ± 16.3	95.4 ± 16.9	.005	98 ± 16.8	97.1 ± 16.8	.551	97.2 ± 16.7	95.8 ± 16.9	.297	98.2 ± 15.1	97.8 ± 18.3	.884
Postbronchodilator FVC%	101.3 ± 13.6	99.5 ± 14.2	.060	101 ± 12.3	101.9 ± 14.5	.431	100.8 ± 13.7	100.1 ± 15	.568	103.3 ± 13.5	102.7 ± 15.1	.811
Postbronchodilator FEV_1_/FVC	0.79 ± 0.09	0.77 ± 0.09	.010	0.77 ± 0.09	0.76 ± 0.09	.045	0.77 ± 0.09	0.77 ± 0.09	.326	0.76 ± 0.08	0.75 ± 0.1	.794
FEV_1_ bronchodilator response	5.7 (6.6)	6.5 (5.7)	.053	7.6 (8.5)	9.3 (8.9)	.023	6.8 (6.8)	7.6 (7.3)	.164	11.1 (10.4)	11.5 (12.8)	.844
Positive bronchodilator responsiveness test	30 (7.2)	57 (12.8)	.010	30 (16.3)	136 (26.1)	.009	161 (13.3)	32 (16.9)	.243	33 (35.5)	27 (38)	.796
Allergy												
Allergic sensitization	187 (44.8)	208 (46.7)	.676	88 (47.8)	305 (58.5)	.023	593 (48.8)	93 (49.2)	.999	59 (63.4)	43 (60.6)	.444
Clinical allergy	177 (42.4)	191 (42.9)	.986	83 (45.1)	297 (57)	.002	557 (45.9)	90 (47.6)	.823	59 (63.4)	42 (59.2)	.999
Medications												
Any ICS use	182 (43.6)	229 (51.5)	.026	81 (44)	275 (52.8)	.049	566 (46.6)	110 (58.2)	.004	44 (47.3)	47 (66.2)	.024

Continuous variables are shown as mean ± SD, and categorical variables as n (%). Bronchodilator response (%) refers to the percentage increase in FEV_1_ after bronchodilator use. A positive bronchodilator responsiveness test was defined as an increase in FEV_1_ of ≥12% and ≥200 mL. Clinical allergy was defined by the presence of allergic sensitization (positive SPT or sIgE to any allergen), and self-reported allergic symptoms attributable to the same allergen family. Statistical comparisons across the groups were conducted using Student *t* test for continuous variables and Pearson’s chi-square test for categorical variables. *P* < .05 indicate statistical significance.

**Table II:** Participants characteristics, lung function, and allergy stratified by cutoffs of Feno (20-50 ppb) and BEC (300-150 cells/μL) (N = 1,567)

Feno (ppb)	≤20		*P* value	>20		*P* value	≤50		*P* value	>50		*P* value
BEC (cells/μL)	≤300	>300		≤300	>300		≤150	>150		≤150	>150	
Demographics												
No. participants	779 (90.4)	83 (9.6)		528 (74.9)	177 (25.1)		584 (41.6)	819 (58.4)		17 (10.4)	147 (89.6)	
Age (y)	48 ± 15.5	47.6 ± 15.2	.828	51 ± 15.4	52.1 ± 15.8	.408	48.1 ± 15.6	50 ± 15.6	.025	51.3 ± 16.7	51.7 ± 15.2	.927
Sex (male)	247 (31.7)	35 (42.2)	.071	263 (49.8)	84 (47.5)	.649	207 (35.4)	335 (40.9)	.044	13 (76.5)	74 (50.3)	.070
BMI (kg/m^2^)	26.9 ± 5.2	27.2 ± 5.4	.628	26.6 ± 4.2	26.5 ± 4.5	.752	26.5 ± 4.8	27.1 ± 5	.011	26.1 ± 3.4	26.3 ± 4.1	.795
Never smokers	379 (48.7)	35 (42.2)	.303	287 (54.4)	108 (61)	.145	312 (53.4)	399 (48.7)	.080	12 (70.6)	86 (58.5)	.483
Current smokers	98 (12.6)	23 (27.7)	<.001	29 (5.5)	7 (4)	.544	59 (10.1)	89 (10.9)	.711	1 (5.9)	8 (5.4)	.999
Pack-year	14.3 ± 14.4	16.6 ± 16.9	.387	13.3 ± 13.2	12.9 ± 16.7	.858	14.8 ± 15.4	14.2 ± 14.2	.618	10.5 ± 7	9.9 ± 11.3	.890
Lung function												
Prebronchodilator FEV_1_%	92.2 ± 17.5	88.4 ± 16.3	.049	90.6 ± 17.4	90.1 ± 19.9	.766	93.4 ± 17.3	90.1 ± 17.7	<.001	94.2 ± 20.7	88.9 ± 18.5	.327
Prebronchodilator FVC%	98.7 ± 14.5	95.6 ± 16.3	.096	98.7 ± 14.8	98.5 ± 17	.917	99.6 ± 14.2	97.6 ± 15.5	.012	100.8 ± 17.7	99 ± 15.3	.695
Prebronchodilator FEV_1_/FVC	0.75 ± 0.09	0.75 ± 0.07	.649	0.73 ± 0.09	0.72 ± 0.09	.500	0.75 ± 0.09	0.74 ± 0.09	.002	0.73 ± 0.1	0.71 ± 0.09	.365
Postbronchodilator FEV_1_%	97.3 ± 16.8	94 ± 15.8	.077	97.3 ± 16.4	97.5 ± 17.8	.891	98.3 ± 16.5	96.1 ± 16.9	.014	101.7 ± 16.2	97.6 ± 16.6	.339
Postbronchodilator FVC%	100.5 ± 13.7	98.4 ± 15.7	.237	101.6 ± 13.8	101.9 ± 14.7	.763	101.1 ± 13.1	100.3 ± 14.4	.295	102.8 ± 15.1	103.1 ± 14.1	.937
Postbronchodilator FEV_1_/FVC	0.78 ± 0.1	0.77 ± 0.08	.418	0.76 ± 0.09	0.76 ± 0.09	.633	0.78 ± 0.09	0.77 ± 0.09	<.001	0.78 ± 0.08	0.75 ± 0.09	.193
FEV_1_ bronchodilator response	6.1 (6.2)	6.1 (5.5)	.994	8.5 (8.2)	9.8 (10.4)	.141	6.1 (6.9)	7.4 (6.8)	<.001	10.6 (15.3)	11.4 (11)	.850
Positive bronchodilator responsiveness test	77 (9.9)	10 (12)	.692	117 (22.2)	49 (27.7)	.182	56 (9.6)	137 (16.7)	<.001	4 (23.5)	56 (38.1)	.292
Allergy												
Allergic sensitization	361 (46.3)	34 (41)	.333	291 (55.1)	102 (57.6)	.350	265 (45.4)	421 (51.4)	.040	10 (58.8)	92 (62.6)	.244
Clinical allergy	335 (43)	33 (39.8)	.494	281 (53.2)	99 (55.9)	.390	250 (42.8)	397 (48.5)	.026	10 (58.8)	91 (61.9)	.513
Medications												
Any ICS use	366 (47)	45 (54.2)	.255	244 (46.2)	112 (63.3)	<.001	257 (44)	419 (51.2)	.010	6 (35.3)	85 (57.8)	.131

Continuous variables are shown as mean ± SD, and categorical variables as n (%). Bronchodilator response (%) refers to the percentage increase in FEV_1_ after bronchodilator use. A positive bronchodilator responsiveness test was defined as an increase in FEV_1_ of ≥12% and ≥200 mL. Clinical allergy was defined by the presence of allergic sensitization (positive SPT or sIgE to any allergen), and self-reported allergic symptoms attributable to the same allergen family. Statistical comparisons across the groups were conducted using Student *t* test for continuous variables and Pearson’s chi-square test for categorical variables. *P* < .05 indicates statistical significance.

**Fig 2:**
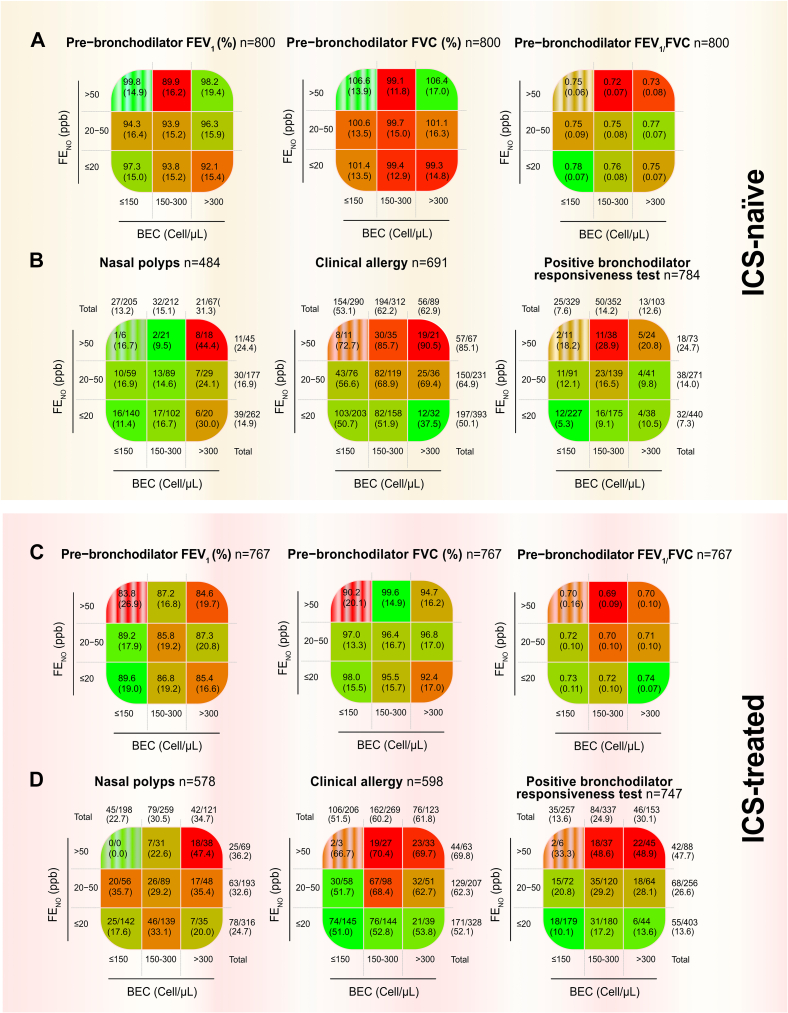
Characteristics of all asthma participants stratified by levels of Feno and BEC. Participants who were ICS-naive (n = 800) and ICS-treated (n = 767) were grouped into 9 strata based on combinations of Feno (≤20, 20-50,and >50 ppb) and BEC (≤150, 150-300, and >300 cells/μL), as shown in heatmap-style plots, heat (*red*) reflecting worse outcome. Each cell presents the mean ± SD or n (%). **(A)** Prebronchodilator lung function: FEV_1_%, FVC%, and FEV_1_/FVC ratio among participants who were off ICS treatment. **(B)** Prevalence of nasal polyps, clinical allergy, and positive bronchodilator responsiveness test (defined as ≥12% and ≥200 mL improvement in FEV_1_ after bronchodilator) among participants who were off ICS treatment. **(C)** Prebronchodilator lung function: FEV_1_%, FVC%, and FEV_1_/FVC ratio among participants who were on ICS treatment. **(D)** Prevalence of nasal polyps, clinical allergy, and positive bronchodilator responsiveness test among participants who were on ICS treatment. Note: Differences in subgroup number of participants are because of the missing data with certain outcome variables. *Striped* colors in the heatmaps indicate low number of participants.

Among individuals who were ICS-naive, participants with Feno >50 ppb and BEC >300 cells/μL did not exhibit the poorest lung function compared with other biomarker-defined groups (Fig 2, *A*). However, this group showed the highest prevalence of nasal polyps and clinical allergy (Fig 2, *B*).

Among individuals who were ICS-treated, participants with Feno >50 ppb and BEC >300 cells/μL did not exhibit the poorest lung as well, but showed the highest prevalence of nasal polyps, clinical allergy, and positive bronchodilator responsiveness test (Fig 2, *C* and *D*).

### Associations of BEC and Feno with lung function and allergic parameters in unselected asthma population

**Fig 3:**
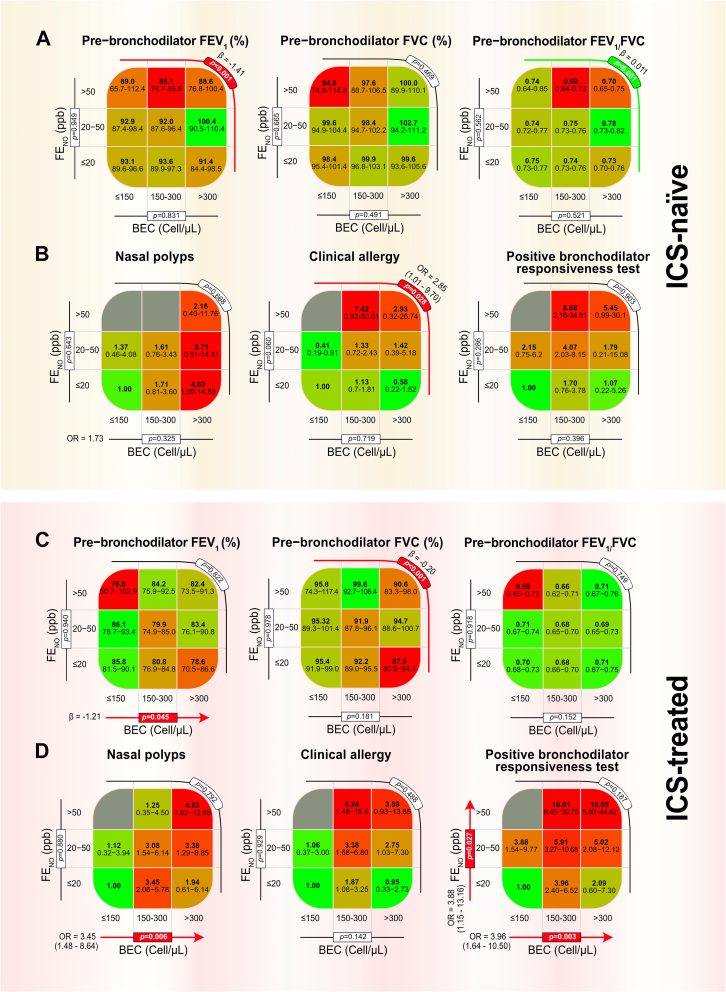
Model-derived characteristics of participants with asthma stratified by Feno and BEC. Participants who were ICS-naive (n = 800) and ICS-treated (n = 767) were categorized into 9 strata based on combinations of Feno (≤20, 20-50, and >50 ppb) and BEC (≤150, 150-300, and >300 cells/μL). Heatmap-style plots display adjusted predicted mean ± SD values for continuous outcomes and adjusted ORs with 95% CIs for binary outcomes, derived from multivariable regression models (linear or logistic). Color gradients reflect the magnitude of the model-predicted estimates, with *red* indicating less favorable and *green* indicating more favorable outcomes from a clinical perspective. *Gray* cells indicate strata where reliable model estimates could not be obtained due to low numbers of participants or outcome events. **(A)** Prebronchodilator lung function (FEV_1_%, FVC%, and FEV_1_/FVC ratio) among participants who were off ICS treatment. **(B)** Adjusted ORs of having nasal polyps, clinical allergy, and positive bronchodilator responsiveness among participants who were off ICS treatment. **(C)** Prebronchodilator lung function (FEV_1_%, FVC%, and FEV_1_/FVC ratio) among participants who were on ICS treatment. **(D)** Adjusted ORs of having nasal polyps, clinical allergy, and positive bronchodilator responsiveness among participants who were on ICS treatment. The y-axis *P* value is for Feno in the regression model. The x-axis *P* value is for BEC in the regression model. The *P* value in the table corner shows the interaction term between Feno and BEC in the regression model. *P* < .05 is considered significant.

In trend analyses using linear and logistic regression models adjusted for age, sex, BMI, and smoking (for continuous and categorical outcomes, respectively), neither Feno nor BEC was independently associated with any outcome among participants who were ICS-naive. However, a significant interaction between Feno and BEC was observed, showing a negative association with FEV_1_% (β = −1.41) and an increased likelihood of clinical allergy (OR = 2.85). A very modest positive interaction effect was also observed for the FEV_1_/FVC ratio (β = 0.011) (Fig 3, *A* and *B*).

Among participants who were ICS-treated, higher BEC was independently associated with lower FEV_1_% (β = −1.21) and a higher prevalence of nasal polyps (OR = 3.45), regardless of Feno levels. In contrast, the Feno –BEC interaction was associated with lower FVC% (β = −0.20). Both Feno and BEC were independently associated with a positive bronchodilator responsiveness test (OR = 3.88 and 3.96), with no evidence of interaction (Fig 3, *C* and *D*).

### Influence of different cutoff values of BEC and Feno on their associations with outcomes

To see whether the associations among BEC and Feno and lung function, nasal polyps, clinical allergy, and positive bronchodilator responsiveness test changes with cutoff values other than the standard ones, we divided BEC into 26 cutoff points, from 100 to 350 cells/μL in steps of 10. Feno was divided into 9 cutoff points, from 15 to 55 ppb in steps of 5. For each cutoff combination, we re-estimated the linear regression model of the continues outcomes or logistic regression model of the binary outcomes. From each model, regression coefficients (β) or OR and corresponding *P* values were extracted for the main effects of Feno and BEC as well as for their interaction, with outcomes. The estimated β coefficients or ORs were plotted across all cutoff combinations to illustrate the direction and magnitude of associations, while the corresponding *P* values were plotted on the secondary y-axis to indicate statistical significance.

**Fig 4:**
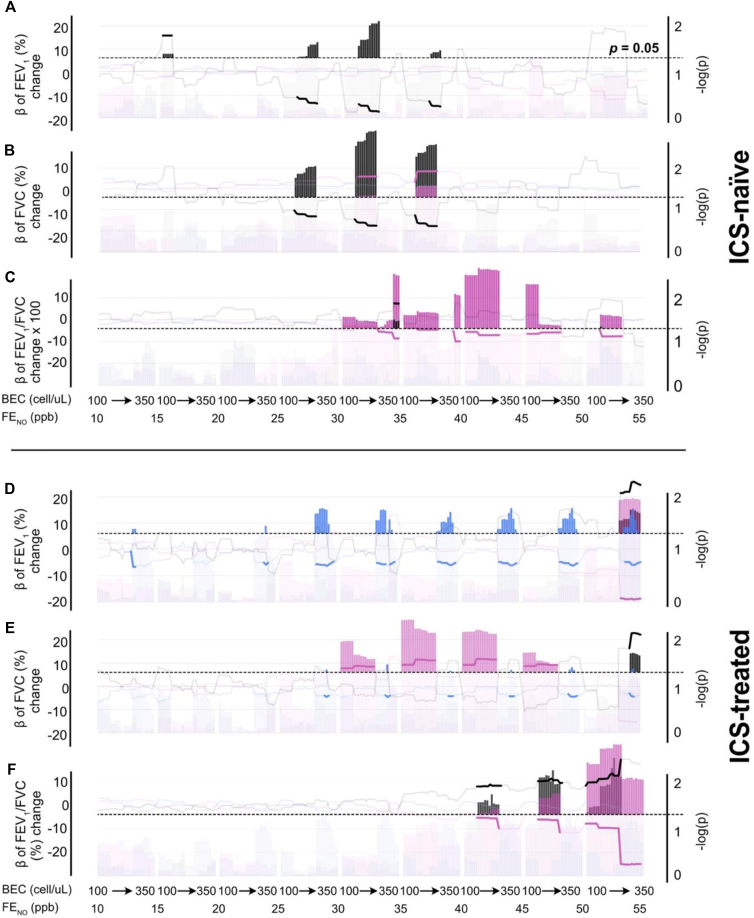
The estimated regression coefficients (β) for the main effects of Feno (*pink*), BEC (*blue*), and their interaction (*black*), derived from multivariable linear regression models at each cutoff combination (*left* y-axis). **(A)** FEV_1_%, **(B)** FVC%, and **(C)** FEV_1_/FVC percentage among participants who were ICS-naive (n = 800), and **(D)** FEV_1_%, **(E)** FVC%, and **(F)** FEV_1_/FVC percentage among participants who were ICS-treated (n = 767). *Colored* lines represent estimated regression coefficients values of lung function parameter on *left* y-axis for each Feno -BEC combinations divides. *Vertical bars* in the background represent the statistical significance of the association of Feno (*pink*)-BEC (*blue*) or their interaction (*black*) at each of the threshold combination, indicated by −log(*P*) of *right* y-axis. The *black dashed line* marks the threshold of *P* = .05 and values above this line indicate statistical significance. No adjustment for multiple testing was made.

Among participants who are ICS-naive, the interaction term of Feno-BEC was associated with lower FEV_1_% and FVC% across all cutoffs of Feno between 25 and 40 ppb and BEC >200 cells/μL (Fig 4, *A* and *B*). When cutoffs of Feno >25 ppb and BEC <300 cells/μL, Feno was independently associated with lower FEV_1_/FVC percentage (Fig 4, *C*).

However, among participants who were ICS-treated, having high BEC was independently associated with lower FEV_1_% and FVC% across all Feno cutoffs >20 ppb. Meanwhile, high Feno was independently associated with having higher FVC% but lower FEV_1_% and FEV_1_/FVC percentage with high cutoffs of Feno. The interaction term of Feno -BEC was associated with higher FEV_1_% and FEV_1_/FVC percentage only with high cutoffs of Feno and BEC (Fig 4, *D*-*F*).

**Fig 5:**
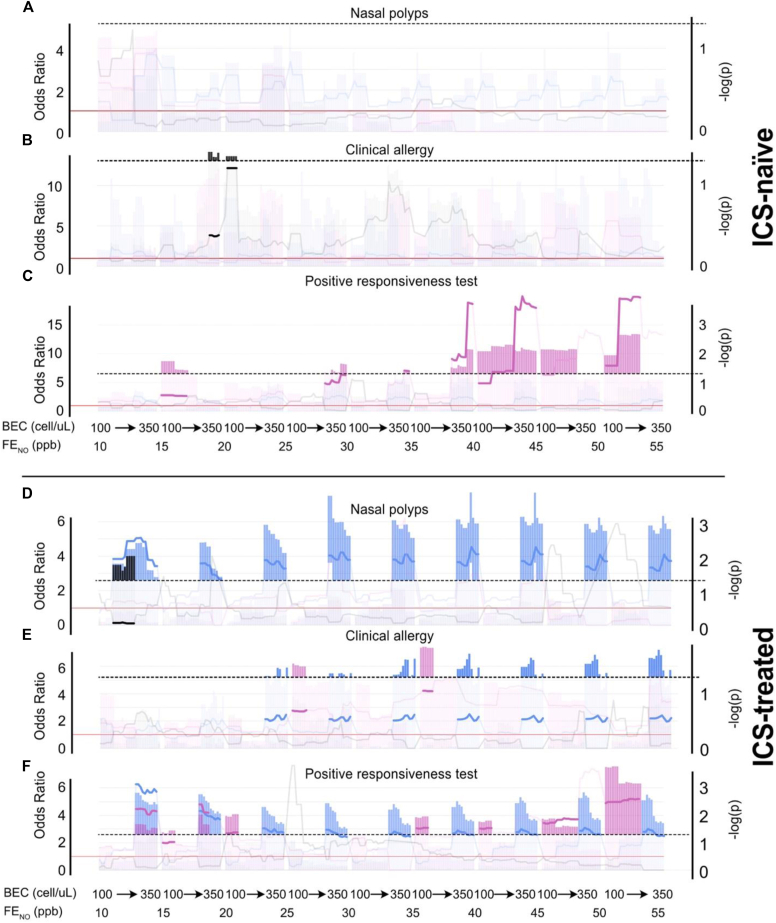
The adjusted ORs for the main effects of Feno (*pink*), BEC (*blue*), and their interaction (*black*), derived from multivariable logistic regression models at each cutoff combination (*left* y-axis). **(A)** Nasal polyps, **(B)** clinical allergy, and **(C)** positive bronchodilator responsiveness test among participants who were ICS-naive (n = 800), and **(D)** nasal polyps, **(E)** clinical allergy, and **(F)** positive bronchodilator responsiveness test among participants who were ICS-treated (n = 767). *Colored lines* represent adjusted ORs values of having each outcome on *left* y-axis for each Feno-BEC combinations divides. *Vertical bars* in the background represent the statistical significance of the association of Feno (*pink*)-BEC (*blue*) or their interaction (*black*) at each of the threshold combination, indicated by −log(*P*) of *right* y-axis. The *black dashed line* marks the threshold of *P* = .05 and values above this line indicate statistical significance. The *red line* marks the threshold of OR = 1. No adjustment for multiple testing was made.

Among participants who were ICS-naive, Feno, BEC, and their interaction term were not associated with having nasal polyps or clinical allergy in almost all cutoff combinations (Fig 5, *A* and *B*). Meanwhile, Feno was independently associated with higher ORs of having positive responsiveness test (Fig 5, *C*). However, among participants who were ICS-treated, having high BEC was independently associated with higher ORs of having nasal polyps, clinical allergy, and positive responsiveness test among almost all cutoff combinations (Fig 5, *D*-*F*). Feno was associated with higher ORs of having positive responsiveness test only with low BEC cutoffs (<300 cells/μL) (Fig 5, *F*). No obvious effect of the interaction term of Feno-BEC was observed.

## Discussion

In this unselected asthma population, the associations of Feno and BEC with lung function and allergic outcomes differed markedly by ICS treatment status. Among participants who were ICS-naive, neither Feno nor BEC showed independent associations with outcomes; instead, their combined effect was most relevant. Specifically, a significant Feno-BEC interaction was associated with lower FEV_1_%, FVC%, and a higher prevalence of clinical allergy, with consistent effects across a range of cutoff values.

**Fig 6:**
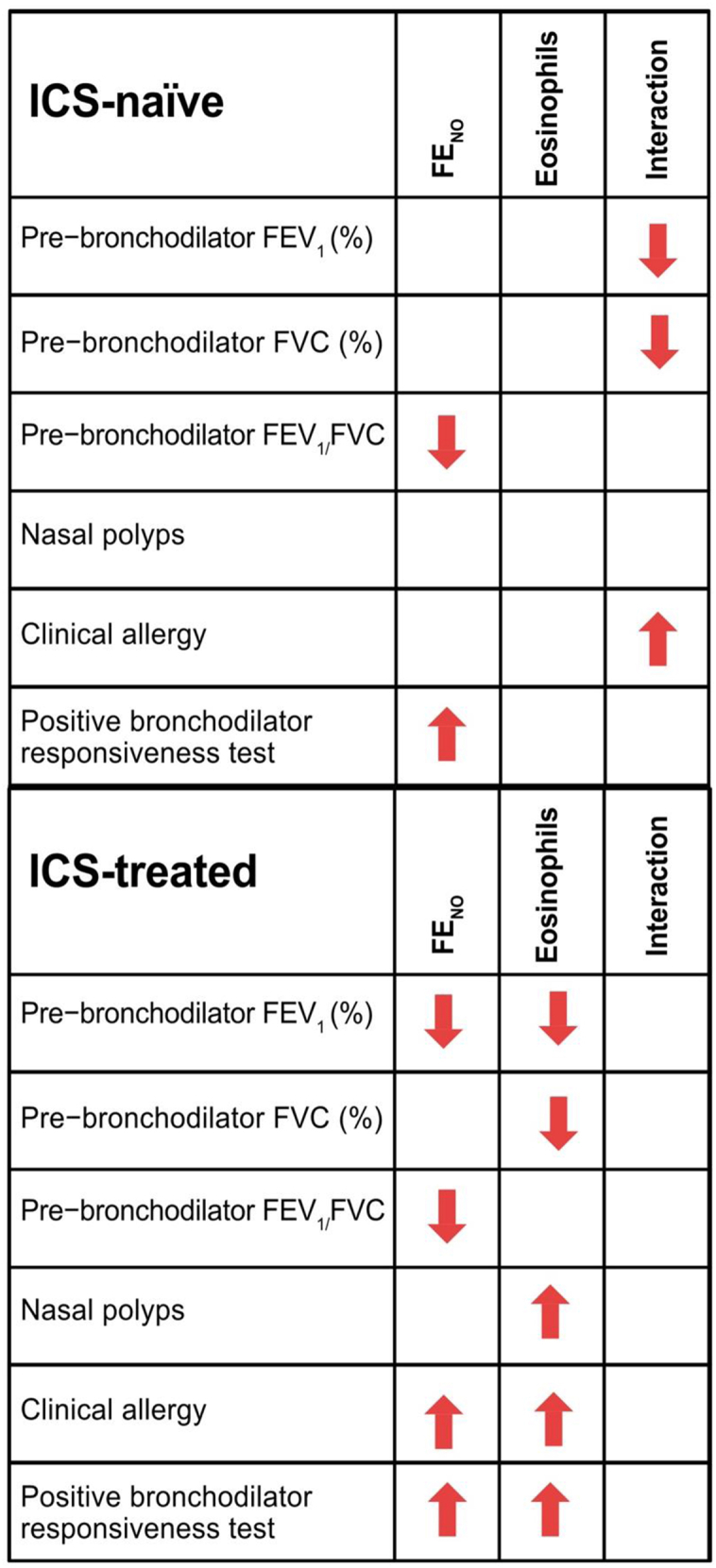
Summary of associations between Feno, BEC, and worsening clinical characteristics across participants who were ICS-naive and those who were ICS-treated.

In contrast, among participants who were ICS-treated, BEC emerged as the dominant biomarker, showing independent associations with reduced lung function, higher nasal polyps, and together with Feno contributed to bronchodilator responsiveness. Interaction effects between Feno and BEC were limited in this group (Fig 6).

Few studies have examined the independent association of T2 inflammation markers—BEC and Feno—on lung function decline, measured by absolute FEV_1_ reduction.^26^, ^27^, ^28^ One study assessed changes in FEV_1_/FVC ratio, FEV_1_%, and FVC%.^5^ From the previous studies, only 1 study^27^ explored the combined association of BEC and Feno on FEV_1_ decline, using a single cutoff (20 ppb for Feno, 300 cells/μL for BEC). Our study is the first to examine how these 2 markers act in synergy across multiple lung function and allergy parameters. We also assess their association using various cutoffs in an unselected asthma population. This analysis is a strong tool to test whether BEC and Feno reflect different aspects of T2 inflammation in lung function. This idea is newly proposed and advances current thinking in the field.^4^^,^^12^

In previous studies, BEC >400 cells/μL was significantly associated with lower FEV_1_% decline^5^ and FEV_1_ absolute decline^29^ in adults with asthma. Tan et al^26^ reported that patients who had chronic obstructive pulmonary disease and BEC ≥300 cells/μL had a steeper FEV_1_ decline over 9 years. In healthy individuals, BEC was linked to FEV_1_ decline over 5 years.^30^ A rise in BEC over 4 years was also associated with greater FEV_1_ decline in the general population.^31^ In line with these findings, we found that BEC was independently associated with lower FEV_1_%, regardless of Feno levels among current ICS users. Importantly, these associations persisted after adjustment for Feno, suggesting that elevated BEC in ICS users may reflect persistent eosinophilic inflammation that is not captured by Feno. Importantly, none of the previous studies included Feno in their models. Our findings, however, extend the previous studies’ observations by demonstrating that the association is independent of Feno. Taken together, these results raise the possibility that, in ICS-treated asthma, elevated BEC may signal residual eosinophilic inflammation that is either corticosteroid-resistant or driven by pathways beyond classical IL4/IL13-mediated T2 inflammation. In fact, the observation that higher Feno and BEC cutoffs were each independently associated with lower lung function among participants who were ICS-treated, while their interaction was associated with better lung function at high cutoffs. This could be that Feno and BEC in isolation reflect different forms of residual or uncontrolled inflammation under ICS therapy. Elevated BEC may indicate persistent systemic eosinophilic inflammation that is relatively corticosteroid-resistant or driven by non-IL4/IL13 pathways, whereas elevated Feno may reflect ongoing epithelial NO production related to local airway inflammation or poor ICS adherence. When both biomarkers are elevated simultaneously, however, this may identify a subgroup with a more “classical” and coherent T2 inflammatory phenotype that remains at least partially responsive to corticosteroids, resulting in relatively preserved lung function despite high biomarker levels. In this context, patients with concordantly high Feno and BEC represent individuals with active but treatment-responsive inflammation, whereas discordant elevation (high Feno or high BEC alone) may signal more heterogeneous, non-T2–dominant, or corticosteroid-insensitive disease processes associated with worse lung function.

Studies have found associations between Feno and FEV_1_ decline over 3 years,^32^ 5-6 years,^33^ and up to 20 years in patients with asthma.^34^ However, these studies did not assess the interaction association of Feno and BEC. Colak et al.^27^ found that patients with asthma and high BEC had faster FEV_1_ decline. The decline was even greater in those with high Feno. In their study, patients with both biomarkers elevated had the largest decline. However, these studies did not clarify whether the link between Feno and lung function was due to independent contributions of BEC and Feno, or an interaction between them or basically due its correlation with BEC,^35^ especially when some studies found no such association.^36^^,^^37^ In participants not receiving ICS treatment in our study, neither BEC nor Feno showed independent associations with lung function or allergic outcomes when modeled together. Instead, their interaction emerged as the dominant signal. Specifically, the Feno-BEC interaction was associated with lower FEV_1_% and a higher prevalence of clinical allergy, with consistent effects across commonly used and alternative cutoff values. These findings suggest that, in the absence of ICS, Feno and BEC reflect complementary aspects of active T2 inflammation, with combined elevation identifying individuals with greater functional impairment and allergic burden.

The associations of BEC and Feno on lung function decline, along with other covariates, have been analyzed using 2 multivariable regression models. Graff et al^38^ reported that only BEC—not Feno—was strongly linked to lung function decline over 5 years in patients with asthma. Coumou et al^28^found both Feno and BEC were associated with faster decline in postbronchodilator FEV_1_, but not in FEV_1_%, in newly diagnosed asthma cases. Our results incorporate both findings. We show that BEC was independently associated with lower FEV_1_%, regardless of Feno levels among current ICS users. In contrast, Feno was independently associated with bronchodilator responsiveness. This pattern suggests that Feno may primarily reflect ongoing airway epithelial inflammation and corticosteroid-responsive NO signaling, rather than structural or persistent inflammatory processes driving lung function impairment. The dissociation between Feno and BEC in individuals who were ICS-treated supports the notion that these biomarkers capture distinct biological processes once anti-inflammatory therapy is introduced.

BEC was a strong marker for nasal polyps in our study among ICS users. This association did not interact with Feno levels. Meanwhile, Feno alone was not a strong marker of nasal polyps. In patients with severe asthma, both BEC and Feno were associated with nasal polyposis and chronic rhinosinusitis with nasal polyps,^39^^,^^40^ Maniscalco et al^39^ found that although both markers were associated with increased risk, BEC had a stronger association than Feno, both statistically and in terms of OR. In their sensitivity analysis, they stratified patients by BEC levels (high vs low). Those with high BEC had 14.5 times higher odds of having nasal polyps than those with low BEC (. < .001). In contrast, among those with low BEC, high Feno was associated with only 5.6 times higher odds with *P* = .03. Nevertheless, our finding that Feno is not associated with polyps is somewhat surprising as blockage of the IL4R/IL13 pathway by dupilumab has good efficacy against polyps.^41^ However, this discrepancy may reflect differences between systemic eosinophilic burden and localized airway cytokine activity, or the modifying effects of ICS on Feno levels.

In our study, both BEC and Feno were interactive markers of clinical allergy. In patients with severe asthma, the percentage of those with high IgE and high Feno, was higher than those with high IgE and BEC.^42^ In an unselected population of individuals with severe asthma, 8% had high IgE and high Feno, and another 8% had high IgE and high BEC. Fifteen percent had all 3 markers elevated.^1^ Among those with high IgE and high Feno, 93% showed allergic sensitization. In addition, 46% of those with high IgE and high BEC were sensitized. These results suggest that both BEC and Feno are associated with higher IgE.

A major strength of this study is its large, population-based design that includes a broad and unselected asthma cohort, which enhances the generalizability of the findings. In addition, rigorous and standardized clinical assessments, including spirometry, Feno, BEC, sIgE, and SPT, were conducted following international guidelines. The use of multiple biomarker cutoffs and interaction modelling adds another dimension to our understanding of T2 inflammation. This is important given that BEC and Feno are correlated, and this could contribute to confounding outcomes of many studies that do not reflect on the interaction of these 2 markers. Despite the abovementioned strengths, this study has limitations. First, its cross-sectional design limits the ability to infer causality between biomarker levels and lung function outcomes. Second, some subgroups defined by extreme biomarker levels were relatively small, potentially limiting the power to detect subtle interactions.

The key clinical and novel finding of our study is the exploration of distinct interactions among classic T2 inflammation markers in relation to lung function, nasal polyps, and allergy with and without ICS treatment. In an ICS-naive unselected asthma population, BEC was associated with reduced lung function (lower FEV_1_%), interacting with Feno. This suggests that eosinophilic inflammation driven by T2 inflammation contributes to worse outcomes of lung function and that BEC and Feno have a synergistic association in this context possibly related to heavy activations of different compartments of T2 inflammation pathway. This aligns with previous findings in severe asthma, where both biomarkers offer prognostic (for attacks) and predictive (for treatment) value.^14^^,^^43^ In contrast, FEV_1_ reversibility was mainly linked to Feno especially among ICS users where interaction effects between Feno and BEC were limited in this group. This suggests that Feno is a stronger marker of local airway inflammation and that each biomarker represents a distinct compartment of inflammation, as previously suggested^12^: BEC mainly relates to lung function decline and inflammation, while Feno reflects airflow limitation and reversibility. These findings support the current guidelines in their recommendation to assess both BEC and Feno to phenotype patients with asthma (and maybe chronic obstructive pulmonary disease).

### Conclusion

In untreated asthma, Feno and BEC act synergistically, reflecting active and coordinated T2 inflammation associated with impaired lung function and allergic disease. In contrast, among ICS users, elevated BEC appears to mark persistent eosinophilic inflammation and structural disease manifestations, such as reduced lung function and nasal polyps, that are less dependent on Feno. These results underscore the importance of measuring both biomarkers in asthma and suggest that elevated BEC in patients who were ICS-treated may identify a subgroup with ongoing eosinophilic disease requiring alternative or additional therapeutic strategies.

**Key messages**

• T2 inflammation markers (Feno and BEC) may reflect different T2 pathways, and their associations with different asthma characteristics in unselected populations remain unclear.
• The synergistic effect of Feno and BEC was associated with lower FEV_1_% and FVC%, and a higher prevalence of clinical allergy among participants who were ICS-naive.
• BEC, independently of Feno, was associated with reduced FEV_1_% and FVC%, as well as higher nasal polyps and clinical allergy among participants who were ICS-treated.

## Disclosure statement

The study was supported by the VBG Group Herman Krefting Foundation for Asthma and Allergy Research (Trollhättan, Sweden), Swedish Research Council (Stockholm, Sweden), the Swedish Heart-Lung Foundation (Stockholm, Sweden), the Swedish Asthma and Allergy Foundation (Stockholm, Sweden), Tampere Tuberculosis Foundation (Tampere, Finland), and Avtal om Läkarutbildning och Forskning (ALF) agreement (grant from the Swedish state under the agreement between the Swedish Government and the county councils, Sweden).

Disclosure of potential conflict of interest: R. Abohalaka reports grants to attend international meetings from the European Respiratory Society (ERS), the Swedish Heart and Lung Foundation (HLF), the Adlerbertska foundations, and the American Thoracic Society (ATS) outside the current work. L. Lehtimäki reports personal fees from ALK-Abelló, AstraZeneca, Berlin Chemie, Boehringer-Ingelheim, Chiesi, GSK, Menarini, Orion Pharma, and Sanofi outside the current work. S.S. Özuygur Ermis reports conference-attendance related costs from Thermo Fisher Scientific outside the current work. P. Ilmarinen is employed by GSK as medical advisor. H. Backman reports personal fees for Chiesi for Advisory Board outside the current work. M. Rådinger reports fees for lectures from AstraZeneca and GSK. B.I. Nwaru reports personal fees for lectures and consulting from DBV Technologies and AstraZeneca outside the current work. H. Kankaanranta reports fees for lectures and/or consulting from AstraZeneca, Boehringer-Ingelheim, Chiesi, Covis Pharma, GSK, MedScape, MSD, Orion Pharma, and Sanofi outside the current work. The rest of the authors declare that they have no relevant conflicts of interest.

## Supplementary data

Supplementary Table E1
