# Supplementary material for: Fractional exhaled nitric oxide and blood eosinophils in relation to lung function, allergy and nasal polyps in asthma
Source: J Allergy Clin Immunol Glob. 2026 Jun 1;5(5):100739. doi: 10.1016/j.jacig.2026.100739 (PMC13315671; doi:10.1016/j.jacig.2026.100739)
Supplement: Supplementary Table E1 [file mmc1.docx]

**Fractional Exhaled Nitric Oxide and Blood Eosinophils in Relation to Lung Function, Allergy and Nasal Polyps in asthma**

Reshed Abohalaka^a^ MSc, Lauri Lehtimäki^b,c^ MD PhD, Selin Ercan^a^ MD, Daniil Lisik^d,a^ MD, Saliha Selin Özuygur Ermis^a^ MD, Pinja Ilmarinen^c,e^ MD PhD, Teet Pullerits^a^ MD PhD, Helena Backman^d^ PhD, Madeleine Rådinger^a^ PhD, Bright I. Nwaru^a^ PhD, Hannu Kankaanranta^a,c,e^ MD PhD

^a^ Krefting Research Centre, Department of Internal Medicine and Clinical Nutrition, Institute of Medicine, Sahlgrenska Academy, University of Gothenburg, Gothenburg, Sweden

^b^ Allergy Centre, Tampere University Hospital, Tampere, Finland

^c^ Faculty of Medicine and Health Technology, Tampere University, Tampere, Finland

^d^ Department of Public Health and Clinical Medicine, Umeå University, Umeå, Sweden

^e^ Department of Respiratory Medicine, Seinäjoki Central Hospital, Seinäjoki, Finland

Corresponding author: Reshed Abohalaka

Krefting Research Centre

Department of Internal Medicine and Clinical Nutrition

Institute of Medicine

Sahlgrenska Academy

University of Gothenburg, 41390 Gothenburg, Sweden

Tel: +46 31 786 67 12

E-Mail: reshed.abohalaka@gu.se

Supplement:

Methods

**Study area and population**

The West Sweden Asthma Study (WSAS), a large population-based cohort study, has been described in detail previously (20). Briefly, the study includes participants from two phases: In 2008, 30,000 individuals from the general population were randomly selected and invited to complete a postal survey. A total of 18,087 (60%) responded. From these, 2,000 were randomly chosen and invited for detailed clinical investigations, of which 1,172 participated. Asthma was identified either by self-reported physician diagnosis or based on clinical evaluation at the study center. Among the participants, 188 had asthma. In addition, among the rest of the responders, all those who had reported physician-diagnosed asthma were invited for clinical investigations. A total of 834 of them participated.

In 2016, a new, non-overlapping random sample of 50,000 individuals was invited to complete a postal survey. A total of 24,534 (49%) responded. A random sample of 5,000 responders was invited for clinical investigations. Among them, 171 participants with asthma completed investigations before March 1, 2020. Additionally, among the rest of the responders, all those who reported physician-diagnosed asthma were invited, and 562 of them participated before March 1, 2020. Additionally, individuals who had responded to the 2008 survey were also invited to a follow-up postal survey in 2016. A total of 12,449 completed the follow-up. All those who reported physician-diagnosed asthma in 2016, but not in 2008, were also invited for clinical investigations, and 95 participants successfully completed this step. In the current study, we combined all asthma participants from the previous studies (188 + 834 + 171 + 562 + 95). This resulted in a total of 1,850 unique asthma participants. Participants missing BEC or FE_NO_ measurements (*n*=283) were excluded from the analysis. Thus, a total of 1,567 individuals with asthma and complete biomarker data were included.

**Clinical examinations**

The examinations included, but were not limited to, blood cell quantification, skin prick testing (SPT), specific immunoglobulin E (sIgE) level assessment, spirometry, and measurement of height and weight. Additionally, structured interviews and administration of questionnaires pertaining to respiratory diseases and symptoms, morbidities, healthcare utilization, and potential risk factors were conducted.

**Assessment of blood eosinophils**

BECs were determined as previously described (16) using standard procedures at Sahlgrenska University Hospital (Gothenburg, Sweden) with ADVIA^®^ 2120i Hematology System (Siemens Healthineers, Erlangen, Germany), and are reported as the number of cells per microliter (μL).

**Measurement of FE_NO_**

FE_NO_ was assessed using an electrochemical device (NiOX VERO®, Aerocrine, Morrisville, NC, USA). FE_NO_ measurements were conducted prior to spirometry, and the participants were asked to avoid eating and using tobacco products for 2 hours before the measurements. The analyser sensor was replaced after 300 measurements or one year, whichever came first. All procedures adhered to the latest ATS/ERS guidelines (21, 22). During the measurement, subjects exhaled against a mouth pressure of 5 cm H_2_O aiming at a flow rate of 50 mL/s. NO concentration was measured during the last 5 seconds of a 10-seconds period when flow rate was between 45 and 55 ml/s. If the FE_NO_ measurement did not meet the predefined quality criteria (21, 22), the measurement was repeated until an acceptable result was obtained.

**Measurement of spirometry**

Spirometry tests were done using the MasterScope spirometer (Jaeger, Höchberg, Germany) following ATS/ERS Taskforce guidelines (23, 24). Calibration was performed according to the device manual. A standard volume calibration was done daily using a 3-liter syringe with regular pump strokes. Participants were told not to exercise hard, smoke, or use snus for at least one hour before the test. They were also asked to stop certain medications before the visit. Antihistamines and montelukast were stopped three days before. Long-acting beta-agonists, long-acting muscarinic antagonists, and their combinations were stopped at least 24 hours before. Short-acting beta-agonists were stopped at least 8 hours before. However, participants were allowed to continue inhaled corticosteroids if needed. Reference values from the Global Lung Initiative were used to calculate the Forced Expiratory Volume in 1 second as a percentage of predicted normal (FEV1%) and Forced Vital Capacity as a percentage of predicted normal (FVC%) (25).

Reversibility testing was performed using 4 doses of salbutamol (4x 100 ug = 400 ug) given 15 minutes after the pre-bronchodilator spirometry. A positive reversibility test is defined as an increase in FEV1 of ≥12% and ≥ 200 ml after bronchodilator use. Those who underwent methacholine challenge test (MCT) were excluded from calculation of post-bronchodilator values.

**Assessment of sensitization and clinical allergy**

Sensitization was assessed through the determination of sIgE levels and/or skin prick tests for 11 aeroallergens. In summary, blood samples were procured during clinical visits and subsequently preserved at -80°C. An evaluation of IgE levels against a composite of aeroallergens (Phadiatop) was then undertaken. Individuals exhibiting titers of ≥0.35 kUA/L underwent supplementary measurements for IgE antibody levels against specific allergens within the composite mixture, including cat, dog, horse, house dust mite (Dermatophagoides pteronyssinus, Dermatophagoides farinae), mold (Cladosporium herbarum), birch, timothy grass, and mugwort. Quantification of IgE levels was executed using the ImmunoCAP™ system (Phadia AB, Uppsala, Sweden), where IgE values equal to or surpassing 0.35 kUA/L for an individual allergen were regarded as positive. The SPTs comprised a standard panel of 11 aeroallergens (ALK, Hørsholm, Denmark), administered after a minimum antihistamine withdrawal period of 72 hours. A positive result was defined as a mean wheal diameter ≥3 mm after 15 min. Clinical allergy was defined by the presence of allergic sensitization (positive SPT or sIgE to any allergen; allergic sensitization), and self-reported allergic symptoms attributable to the same allergen family. These symptoms, evaluated during the clinical interview prior to the sensitization test results, included ocular manifestations, nasal discomfort, various forms of allergic nasal expressions, pruritus in the oral or pharyngeal region, respiratory challenges, exacerbation of asthma symptoms, pruritic skin rash, and disruptions in gastrointestinal function.

**Results:**

**Table S1:** Characteristics of WSAS participants (N=1,567).

| **Demograghics** | All | Off ICS treatment | On ICS treatment | *p*-value |
| --- | --- | --- | --- | --- |
| N (%) | 1,567 | 800 (51.0%) | 767 (49.0%) |  |
| Age (year) | 49.4 (15.6) | 46.5 (15.3) | 52.6 (15.3) | **<0.001** |
| Sex (male) | 629 (40.1%) | 352 (44.0%) | 277 (36.1%) | **0.002** |
| BMI (kg/m^2^) | 26.8 (4.8) | 26.4 (4.6) | 27.2 (5.0) | **<0.001** |
| Never smokers | 809 (51.7%) | 418 (52.2%) | 391 (51.0%) | 0.614 |
| Current smokers | 157 (10%) | 89 (11.1%) | 68 (8.9%) | 0.160 |
| Pack-year | 14 (14.4) | 12.8 (13.4) | 15.2 (15.3) | **0.033** |
| **Lung Function** |  |  |  |  |
| Pre−bronchodilator FEV1 (%predicted) | 91.3 (17.7) | 95.0 (15.6) | 87.3 (19.0) | **<0.001** |
| Pre−bronchodilator FVC (%predicted) | 98.5 (15.0) | 100.5 (13.9) | 96.4 (15.8) | **<0.001** |
| Pre−bronchodilator FEV1/FVC | 0.74 (0.09) | 0.76 (0.08) | 0.72 (0.10) | **<0.001** |
| Post−bronchodilator FEV1 (%predicted) | 97.1 (16.7) | 100.1 (15.2) | 94.1 (17.7) | **<0.001** |
| Post−bronchodilator FVC (%predicted) | 100.9 (13.9) | 102 (13.3) | 99.7 (14.5) | **0.001** |
| Post−bronchodilator FEV1/FVC | 0.77 (0.09) | 0.79 (0.08) | 0.75 (0.10) | **<0.001** |
| FEV1 bronchodilator response (%) | 7.3 (7.6) | 6.0 (6.0) | 8.8 (8.8) | **<0.001** |
| Positive bronchodilator responsiveness test | 253 (16.5%) | 88 (11.0%) | 165 (21.5%) | **<0.001** |
| **Allergy** |  |  |  |  |
| Allergic sensitization | 788 (65.8%) | 424 (53.0%) | 364 (47.5%) | 0.861 |
| Clinical allergy | 748 (58%) | 404 (50.5%) | 344 (44.9%) | 0.776 |
|  |  |  |  |  |

Continuous variables are shown as mean (SD), and categorical variables as n (%). Bronchodilator response (%) refers to the percent increase in FEV_1_ after bronchodilator use. A positive bronchodilator responsiveness test was defined as an increase in FEV_1_ of ≥12% and ≥200 mL.
